# Did Cognitive Attentional Syndrome Symptoms Predict Stress- and Trauma-Related Symptoms in the Initial Phase of the COVID-19 Pandemic? Results from a Two-Wave Study on a Sample of Polish Internet Users

**DOI:** 10.3390/brainsci13081162

**Published:** 2023-08-03

**Authors:** Małgorzata Dragan, Piotr Grajewski

**Affiliations:** Faculty of Psychology, University of Warsaw, 00-183 Warsaw, Poland; p.grajewski2@uw.edu.pl

**Keywords:** Cognitive Attentional Syndrome, COVID-19 pandemic, stress- and trauma-related disorders, psychopathology, adjustment disorder

## Abstract

Background: According to metacognitive theory, Cognitive Attentional Syndrome (CAS) is a transdiagnostic factor and a main mechanism of psychopathology maintenance. The main goal of this study is to examine whether CAS predicted stress- and trauma-related symptomatology in the first months of the COVID-19 pandemic and three months later. Methods: Initially, 1792 participants were recruited online via social media; the data were collected at two time points. The measures included the Cognitive Attentional Syndrome Questionnaire, the Adjustment Disorder—New Module 20, the International Trauma Questionnaire, and additional measures. Results: Structural equation modeling was conducted in order to determine the relations between the reported stressors, CAS, and symptomatology. At both time points, CAS was a significant mediator between the stressors and symptoms of adjustment disorder. Despite the decrease in the intensity of adjustment disorder symptoms between waves, it was a significant predictor of other psychopathologies at both time points, except for traumatic stress. Conclusions: The findings confirm the assumption that CAS is a transdiagnostic factor of psychopathology and has a mediating role in the relationship between stressors and adjustment disorder and co-occurring symptomatology. The effect was particularly significant in the initial phase of the pandemic, which was highly stressful for many people.

## 1. Introduction

Cognitive Attentional Syndrome (CAS) is a core concept in the theoretical model of metacognitive therapy for psychological disorders [1]. The self-regulatory executive function model (S-REF) [2,3] considers inflexible and recurrent patterns of thinking in response to negative thoughts, feelings, and beliefs as the proximal cause of such disorders. CAS is a set of symptoms associated with this maladaptive style of processing and is considered a transdiagnostic feature of psychopathology. It consists of perseverative and negative thinking (worry or rumination), threat monitoring, and unhelpful and paradoxically inefficient coping strategies used to deal with the first two aspects, such as thought suppression or avoidance.

CAS is hypothesized to develop due to underlying maladaptive metacognitions, which include positive and negative beliefs about thinking (i.e., metacognitive beliefs) [1,4,5]. For example, holding the belief that worrying keeps one safe (positive metacognitive belief) predisposes a person to worrying in response to a negative thought about potential danger. Furthermore, holding the belief that worrying will ruin one’s health (negative metacognitive belief) leads to a persistence of worrying because the person uses unhelpful strategies such as thought suppression to interrupt the process of worrying. The S-REF model posits that in all situations in which there is a discrepancy between the self-relevant goals (outer circumstances and mental states) and perceived goals, the self-regulatory executive function becomes activated. This process might be accompanied by CAS activation and, therefore, negative emotions, self-appraisal, and a sense of threat. While in most people, periods of CAS activation are brief or non-existent, some will experience a vicious circle of prolonged CAS activation. For example, a person with health-related anxiety during the COVID-19 pandemic engages in negative repetitive thoughts about health-related issues due to positive metacognitive beliefs (e.g., “thinking about it will help me prepare”). Consequently, this person may find that they are devoting excessive amounts of time to worrying. This observation may trigger worrying about the act of worrying due to negative metacognitive beliefs about uncontrollability and the negative consequences of worrying (e.g., “I cannot stop these thoughts and I will lose my mind”). Strategies such as attempting to suppress thoughts will probably lead to greater preoccupation with these thoughts, meaning that CAS is likely to continue to occur.

This process is understood in the S-REF model as the cause of emotional and other psychiatric disorders and their common core component. Numerous studies confirm that maladaptive metacognitive beliefs and symptoms of CAS are positively associated with a wide range of psychological and behavioral problems, including emotional disorders such as depression, anxiety, and stress, as well as trauma-related disorders such as adjustment disorder (AjD) and post-traumatic stress disorder (PTSD) [6,7,8,9].

The aim of this study is to examine the association between the symptoms of CAS and stress- and trauma-related psychopathology in the context of the COVID-19 pandemic, which is perceived as a highly stressful situation by most people [10], causing multiple stressors, illnesses, deaths, and strain on healthcare and economic systems. To date, there have been many publications on the prevalence of emotional disorders obtained in studies conducted during the pandemic. For example, in a web-based survey conducted by Rossi et al. [11], the rates of different mental health outcomes were assessed in the Italian general population three to four weeks into the implementation of lockdown measures after the outbreak of the pandemic. Almost a quarter (23%) of the respondents reported symptoms of adjustment disorder, 37% reported post-traumatic stress symptoms, 22% reported symptoms of high perceived stress, 21% reported symptoms of anxiety, and 17% reported symptoms of depression. In many other studies conducted in the first and subsequent months of the pandemic, increased rates of anxiety, depressive symptoms, and stress-related symptoms were found as well [12,13,14,15]. These symptoms seemed to persist throughout the pandemic. Given the wide range of mental health symptoms observed as outcomes of the pandemic, we decided to test whether CAS is a transdiagnostic factor and whether it is the main mechanism behind the persistence of psychopathology according to the metacognitive-theory-predicted symptoms of various emotional disorders in the first months of the pandemic or three months later. In particular, we focused on the symptoms of adjustment disorder and traumatic stress, but additionally focused on other symptoms that were frequently included in studies on mental health during the pandemic (i.e., generalized anxiety and depression).

## 2. Materials and Methods

### 2.1. Participants

The minimum sample size was determined based on a power analysis simulation study [16]. The total sample for this study consisted of 1792 participants who were recruited online via social media (Facebook). They participated voluntarily, and no financial or material reward was offered. Ethical approval for the study was granted by the Ethics Committee of the Faculty of Psychology at the University of Warsaw. The study was conducted via the internet using the Qualtrics platform. Participants had to be 18 years of age or older at the time of the survey; a total of 50 people were excluded from the study because they were under 18 years of age, and 51 were excluded due to partial completion of the questionnaires, so the analysis ultimately included 1693 participants. No other exclusion criteria were applied. The majority of respondents (78%) were female (21% men, and 1% intergender or non-binary), and the mean age of the sample was 31.23 years (SD = 9.60; range: 18–78 years of age; women: M = 31.11, SD = 9.34; men: M = 31.89, SD = 10.22; other: M = 23.00, SD = 3.69).

The data were collected at two time points. The first wave took place from 25 March to 30 April 2020, three weeks after the identification of the first coronavirus-infected patient in Poland, at the time when the state of the epidemic had just been announced and the lockdown restrictions were introduced. It was therefore the time of the greatest restrictions, consisting of, for example, the closure of educational institutions, the closure of borders to air and rail traffic, the prohibition of travel without due need, etc. After consenting, participants completed the online survey, which lasted about 25 min; if they agreed to participate in the second measurement, they were asked to provide an email address. 

The second time point of the study was three months after the first wave; the data were collected from 3 July to 11 August 2020. In total, 418 people responded in the second wave, but only 362 answered questions. As in the first wave, most participants (78%) were female (20% men, and 1% intergender or non-binary), and the mean age of the sample was 30.40 years (SD = 8.39; range: 18–66 years of age). At the beginning of June, the rate of new infections was 300 people per day. In July, however, there was a sharp increase in new infections, with the rate doubling to about 650 infections per day. The rapid increase was related to the ongoing parliamentary elections and increased holiday tourism, during which, among other things, little attention was paid to maintaining social distancing and wearing masks.

### 2.2. Measures

The first part of the online survey included the measurement of sociodemographic variables (gender, age, relationship status, employment status, years of education, and possibility of remote work). Then, respondents provided answers on self-report questionnaires to measure the following symptoms:

Cognitive Attentional Syndrome: The Cognitive Attentional Syndrome Questionnaire (CAS-1) [1,4] consists of 16 items. The first two are questions concerning the frequency of rumination and worry as well as the concentration on threats. A further six items concern maladaptive behaviors used to cope with negative emotions and/or thoughts, such as thought and situation avoidance, drinking or substance abuse, and attempts to control thoughts or emotions. The last eight items concern positive and negative metacognitive beliefs that are core to Cognitive Attentional Syndrome (e.g., “worrying too much could harm me” or “worrying helps me cope”). The results of the questionnaire were calculated in the same way as previous papers [4,17]. The total results can range from 0 to 128, where a higher result indicates a greater level of Cognitive Attentional Syndrome. The Polish version has good psychometric properties; in the current study, the Cronbach’s alpha was 0.82.

Impaired Functioning: The Work and Social Adjustment Scale (WSAS) [18] is a short questionnaire used to measure impaired functioning. It consists of five items with a nine-point scale (0 indicates no impairment at all and 8 indicates severe impairment). Possible scores range from 0 to 45. Based on the results [18], it is possible to distinguish the following three levels of functioning: 1–10 points indicate mild functional impairment; 11–20 points indicate moderately severe functional impairment; and 21+ points indicate severe functional impairment. The original English version of the WSAS was translated into Polish with the use of the back-translation procedure. The Polish version of the scale exhibited satisfactory internal consistency of scores in the current sample (α = 0.80).

Symptoms of Adjustment Disorder: The Adjustment Disorder—New Module 20 (ADNM-20) [19,20] is a questionnaire that measures symptoms of adjustment disorder. The ADNM-20 consists of the following two parts: a list of stressors and an item symptom list. The list of stressful events comprises a wide range of experiences (19 potential stressors, e.g., financial problems, divorce, serious illness) and requires the respondent to reference the event that was the most aggravating in the last six months. For the purpose of this study, the COVID-19 pandemic was also added to this list. The symptom list section measures the symptoms in response to the most distressing event(s) that the respondent has experienced. The ADNM-20 was developed to more closely align with the ICD-11 guidelines for AjD, and this is reflected in its focus on the two core symptom clusters of Preoccupation (four items) and Failure to Adapt (four items) [21]. However, it also includes the four associated symptom clusters of Avoidance (four items), Depression (three items), Anxiety (two items), and Impulsivity (three items). All items are answered on a four-point Likert scale, with possible scores ranging from 20 to 80. The questionnaire consists of the following six subscales: Preoccupation, Failure to Adapt, Avoidance, Depressive Mood, Anxiety, and Impulsivity. Preoccupation and Failure to Adapt are the core symptoms of AjD and can be considered together as one subscale (AjD-C). Avoidance, Depressive Mood, Anxiety, and Impulsivity are accessory symptoms and can also be considered together as one subscale (AjD-AS). The Polish version of the ADNM-20 has excellent internal consistency, with α = 0.95 [10].

Traumatic Stress: The International Trauma Questionnaire (ITQ) [22] is a self-report measure of ICD-11 PTSD symptoms. Respondents complete the ITQ in relation to the most traumatic event they have experienced before answering questions about symptoms. In the current study, they were also given the option of referring to their experience of the COVID-19 pandemic. The PTSD items in the ITQ are completed with reference to how much the respondent has been bothered by each symptom in the past month and are accompanied by three items measuring functional impairment caused by these symptoms. All items are answered on a five-point Likert scale, with possible scores ranging from 0 to 24. A symptom is considered present where a score of ≥2 (moderately) is achieved. PTSD diagnosis requires traumatic exposure, at least one symptom present from each symptom cluster (Re-experiencing, Avoidance, and Sense of Threat), and endorsement of at least one indicator of functional impairment. The psychometric properties of the ITQ were examined in both clinical and general population samples [23]. The measure is available in many language versions, including Polish (www.traumameasuresglobal.com/itq, 20 March 2020). The internal consistency of the PTSD item scores in the current sample was good (α = 0.88).

Depression: Additionally, nine symptoms of depression were measured using the Patient Health Questionnaire-9 (PHQ-9) [24,25], available from the MAPI Research Institute, (www.phqscreeners.com, 20 March 2020). Respondents indicate how often they have been bothered by each symptom over the last two weeks using a four-point Likert scale. Possible scores range from 0 to 27, with higher scores indicating higher levels of depression. A cut-off score of 15 was used to identify participants likely to meet the criteria for depressive disorder, in accordance with the results of the meta-analysis conducted by Manea et al. [26]. The PHQ-9 scores have very good psychometric properties, showing good internal consistency in the current sample (α = 0.88).

Generalized Anxiety: Symptoms of generalized anxiety were measured using the Generalized Anxiety Disorder 7-item scale (GAD-7) [27]. Like the PHQ-9, respondents indicate how often they have been bothered by each symptom over the last two weeks on a four-point Likert scale. Possible scores range from 0 to 21, with higher scores indicating higher levels of anxiety. The cut-off point for the scale is ≥10 points [28]. The GAD-7 has been shown to be a reliable and valid measure in multiple studies; the Polish translation of the scale is available from the MAPI Research Institute (www.phqscreeners.com, accessed on 20 March 2020). The internal consistency of the scores for the current sample was excellent (α = 0.92).

### 2.3. Statistical Analyses

In the first step, descriptive statistics and a series of paired-samples *t*-tests with Hedges’ g [29] were performed using IBM’s SPSS 28. Following the guidelines for managing missing data in longitudinal studies [30], an attrition analysis was performed. Student’s *t*-test was used to investigate differences in the severities of measured symptoms of disorders between the two waves of study. For further investigation, structural equation modeling (SEM) was conducted in Mplus 8.5 [31] in order to determine relations between stressors, Cognitive Attentional Syndrome, impaired functioning, and symptoms of depression, generalized anxiety, AjD, and PTSD. The recommendations of Hu and Bentler [32] were used to determine the data’s goodness of fit to the model. The following parameters were taken into account, in accordance with the most commonly used standards: RMSEA < 0.08 means an acceptable fit and <0.05 means a good fit; CFI > 0.90 and TLI > 0.90 mean an acceptable fit, and CFI and TLI > 0.95 mean a good fit; and SRMR should be <0.08.

## 3. Results

### 3.1. Descriptive Statistics and Attrition Analysis

The descriptive statistics and the prevalence of disorders are shown in Table 1. An attrition analysis was performed using Little’s MCAR test on the variables associated with psychopathology. The percentage of missing data ranged from 78.6% to 88.2%; due to high levels, no data imputation technique was introduced, and partial responses were used for the *t*-test analyses. Little’s MCAR test was insignificant, χ^2^(27) = 24.60, and *p* = 0.579, and we therefore concluded that the missing data were randomly distributed. 

### 3.2. Changes in the Severity of Symptoms

One of the purposes of this study was to examine the changes in the severities of symptoms over time. A series of paired-samples t-tests were conducted to compare the number of stressors and the symptoms of PTSD, adjustment disorder, depression, generalized anxiety, CAS, and impaired functioning between the two time points. There were significant differences in the PTSD scores as follows: Re-experiencing (M^T1^ = 2.93, SD^T1^ = 2.55, M^T2^ = 4.86, SD^T2^ = 2.48, t (286) = −12.86, *p* ≤ 0.001, Hedges’ g = −0.76, 95% CI [−0.90, −0.63]); Avoidance (M^T1^ = 3.77, SD^T1^ = 2.62, M^T2^ = 5.53, SD^T2^ = 2.62, t (286) = −11.91, *p* ≤ 0.001, g = −0.67, 95% CI [−0.80, −0.63]); Sense of Threat (M^T1^ = 3.70, SD^T1^ = 2.72, M^T2^ = 5.37, SD^T2^ = 2.77, t (286) = −11.52, *p* ≤ 0.001, g = −0.61, 95% CI [−0.72, −0.49]); core symptoms of AjD (M^T1^ = 21.65, SD^T1^ = 6.43, M^T2^ = 20.11, SD^T2^ = 6.67, t (329) = 4.91, *p* ≤ 0.001, g = 0.23, 95% CI [0.14, 0.33]); accessory symptoms of AjD (M^T1^ = 31.95, SD^T1^ = 8.98, M^T2^ = 29.98, SD^T2^ = 9.68, t (329) = 4.45, *p* ≤ 0.001, g = 0.21, 95% CI [0.12, 0.30]); Depression (M^T1^ = 11.43, SD^T1^ = 6.73, M^T2^ = 10.05, SD^T2^ = 6.55, t (278) = 4.78, *p* ≤ 0.001, g = 0.21, 95% CI [0.12, 0.30]); Generalized Anxiety (M^T1^ = 9.55, SD^T1^ = 6.10, M^T2^ = 7.71, SD^T2^ = 65.71, t (365) = 4.46, *p* ≤ 0.001, g = 0.31, 95% CI [0.21, 0.41]); and Impaired Functioning (M^T1^ = 20.97, SD^T1^ = 9.12, M^T2^ = 18.23, SD^T2^ = 10.54, t (361) = 5.73, *p* ≤ 0.001, g = 0.28, 95% CI [0.18, 0.37]). The following were statistically insignificant: changes in the number of stressors (M^T1^ = 5.49, SD^T1^ = 2.42, M^T2^ = 5.42, SD^T2^ = 2.50, t (359) = 0.64, *p* = 0.522) and level of Cognitive Attentional Syndrome (M^T1^ = 75.65, SD^T1^ = 20.33, M^T2^ = 73.96, SD^T2^ = 21.23, t (278) = 1.73, *p* = 0.085). The results of this study indicate that there was a statistically significant difference between the severity of symptoms of AjD, depression, generalized anxiety, and impaired functioning between the two time points; their intensities decreased over time. In contrast, the PTSD symptoms worsened between measurements.

### 3.3. Structural Equation Modeling

**Figure 1:**
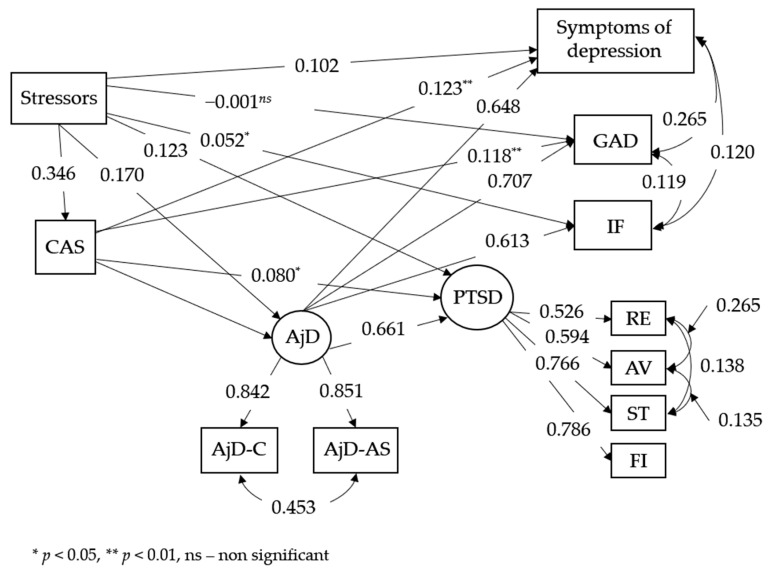
Structural equation model for T1. All paths except those that are marked have *p* < 0.001. Notes: CAS—Cognitive Attentional Syndrome, AjD—adjustment disorder, AjD-C—core symptoms of AjD, AjD-AS—accessory symptoms of AjD, GAD—symptoms of generalized anxiety, RE—re-experiencing, AV—avoidance, ST—sense of rhreat, IF—impaired functioning, FI—PTSD functional impairment, * *p* < 0.05, ** *p* < 0.01, ns—non significant.

The multiple-mediator SEM model was checked using a maximum likelihood estimator (ML) with 1000 bootstrap re-samples. The proposed model of the relationship between the variables at the first measurement (see Figure 1) showed good model fit indicators as follows: χ^2^(28) = 186.155, *p* ≤ 0.001; CFI = 0.984; TLI = 0.969; RMSEA = 0.058 (90% CI [0.050, 0.066]; *p* = 0.049); and SRMR = 0.020. In addition, indirect effects were analyzed between traumatic events, and PTSD included other variables. The results are shown in Table 2. Cognitive Attentional Syndrome and AjD were responsible for a partial mediation in the relationship between the stressors and other studied symptoms.

**Figure 2:**
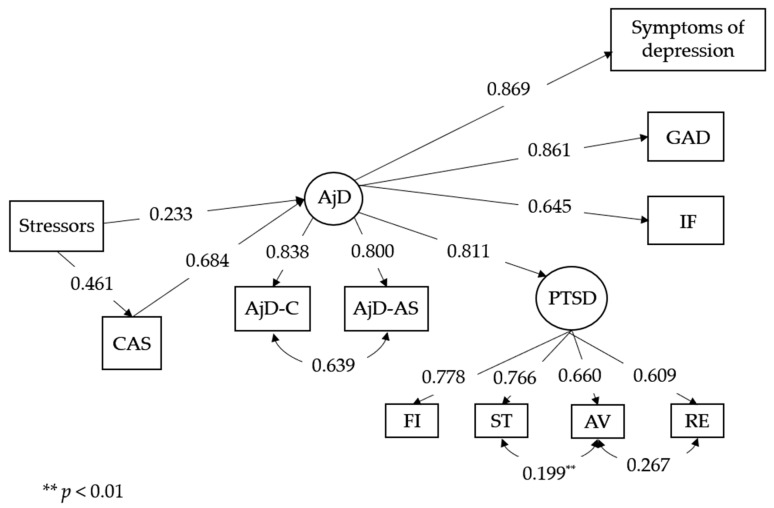
Structural equation model for T2. All paths except those that are marked have *p* < 0.001. Notes: CAS—Cognitive Attentional Syndrome, AjD—Adjustment Disorder, AjD-C—Core Symptoms of AjD, AjD-AS—Accessory Symptoms of AjD, GAD—Symptoms of Generalized Anxiety, RE—Re-experiencing, AV—Avoidance, ST—Sense of Threat, IF—Impaired Functioning, FI—PTSD Functional Impairment, ** *p* < 0.01.

The T1 model was also used for the second measurement. The model fit indicators remained at an acceptable level as follows: χ^2^(28) = 81.748, *p* ≤ 0.001; CFI = 0.976; TLI = 0.952; RMSEA = 0.073 (90% CI [0.055, 0.092]; *p* = 0.020); and SRMR = 0.029. However, not all paths in the model were statistically significant. After removing them (see Figure 2), the model fit indicators improved slightly as follows: χ^2^(39) = 98.023, *p* ≤ 0.001; CFI = 0.973; TLI = 0.962; RMSEA = 0.065 (90% CI [0.049, 0.081]; *p* = 0.061); and SRMR = 0.033. According to the simulations performed on the fit indicators, a value of ∆CFI smaller than or equal to −0.01 indicates that the null hypothesis of invariance should not be rejected [33]. This confirms that the proposed models differ from each other.

## 4. Discussion

According to the metacognitive theory of psychopathology, Cognitive Attentional Syndrome (CAS), with a key feature of perseverative and negative thinking, is the main factor in the development and maintenance of symptoms, including those that appear as a result of an exposure to stress and trauma [10]. This assumption was confirmed in many previous studies. The main aspect of CAS is extended negative thinking in the forms of worry or rumination. In our study, we aimed to answer the question of whether CAS predicted symptoms of stress- and trauma-related disorders in the course of the COVID-19 pandemic, which was perceived as a highly stressful situation by the majority of respondents [10]. This study had a longitudinal design with the following two time points: at the beginning of the pandemic and three months later. Therefore, we analyzed the hypothesized model with reference to these two measurement points that assumed that CAS played a key role in predicting the symptoms of psychopathology.

The first measurement included a large sample of adult Poles recruited through the internet in the first phase of the COVID-19 pandemic (i.e., in the middle of March and April 2020). The tested model revealed that CAS was predictive of all included symptomatology—adjustment disorder, post-traumatic stress disorder, depression, and generalized anxiety. However, it was not predictive of symptoms of impaired functioning measured by the Work and Social Adjustment Scale. The stressors included in the Adjustment Disorder—New Module 20 scale were predictive for CAS, which was a mediator between these events and AjD, PTSD, as well as impaired functioning and depressive symptoms. Cognitive Attentional Syndrome in the first wave was also a direct predictor of all disorders included in this study. In other words, the symptoms of CAS intensified the symptoms of these disorders. This result may be treated as a confirmation of other findings revealing that life stressors are important risk factors for psychopathology in general, but also that AjD is a disorder that is specifically related to stress, because the more stressors there are, the more intense the internal cognitive–emotional processing in the form of worrying/rumination—the core symptom of AjD.

The COVID-19 pandemic, being a new, unknown situation in the global world, has actually been associated with multiple stressors, including deaths due to infection; illnesses; fear of infection; restricted social contact and activity; work-related problems; and many others. However, this complex situation has changed over time and with the number of infections. Therefore, it is worth considering the context of the pandemic at the time the measurements took place. The government of Poland announced the first restrictions on the 11th of March 2020 (the closure of all municipal institutions, schools, and childcare facilities, the banning of all mass events, and even going to the woods), and on March 20th, the state of the epidemic was officially announced. Mandatory government measures followed soon after, with the temporary closure of all non-essential services, additional physical distancing measures, etc. Our study started on the 25th of March—immediately after the introduction of these strict restrictions. Thus, the findings of this study at the first time point can be interpreted in the context of the pandemic as the result of an ongoing process of adaptation to the early phase of this unknown situation, which was somewhat naturally associated with an increased state of stress and anxiety, resulting in an increase in all symptoms of psychopathology.

The pandemic situation was somewhat different at the time of the second measurement; around this time, there was an increase in new infections due to the loosening of the restrictions (July 2020). However, this was also the holiday season, and this significant increase might also be explained by fatigue with the previous strict restrictions that had finally been lifted. The data from that time confirm that CAS mediated between the reported stressors and symptoms of adjustment disorder. However, in this model, it was AjD that mediated the relationship between both stressors and CAS, with co-occurring symptoms of other disorders (PTSD, depression, and generalized anxiety). A significant increase in AjD symptoms predicted the severity of other symptoms, which indicates that intense stress and disturbances in adjustment were dominant features in the picture of psychopathology at that time. This means that extended negative thinking, which is the core aspect of CAS and also one of the main symptoms of AjD, was not only significantly increased, but was also a leading cause of general symptom aggravation. Despite the decrease in the intensity of AjD symptoms between waves, AjD became a significant predictor of other psychopathology symptoms at both time points, except for traumatic stress. The reduced intensity of symptoms may be related to people becoming used to the unique, complex situation and gradually becoming better at coping with it. However, symptoms of PTSD might be specific to other symptoms that co-occur in reaction to prolonged stressful situations or may be increased between two time points as a result of cumulative (traumatic) stress.

The main limitation of this study is, of course, the significant reduction in the number of participants at the second time point, which restricted the possible statistical analyses. Moreover, recruitment in our study relied on voluntary participation through social networks (e.g., Facebook). For this reason, there may be an important selection bias against people who do not use social networks, as well as self-selection, resulting in the highly unbalanced gender ratio (the much higher proportion of women) and the predominance of young adults. An additional factor related to the respondents was a significant representation of people working from home offices. Furthermore, the survey was based on self-report assessments, not interview-based measures. Therefore, the rates of mental health outcomes obtained in this study should be interpreted with caution. Moreover, data on substance use was unfortunately not collected; therefore, no analyses could be performed with substance use disorders as an outcome. Finally, variables that can potentially affect the relationship between CAS and psychopathology, such as fearfulness or anxiety sensitivity, were not included in the study design.

## 5. Conclusions

The results of this study confirm the mediating role of Cognitive Attentional Syndrome between reported stressors and symptoms of emotional disorders (adjustment disorder, traumatic stress, generalized anxiety, and depression) in the context of the initial phase of the COVID-19 pandemic. It also confirms that this situation was highly stressful for many people, as indicated by the symptoms of stress- and trauma-related disorders; however, even in the initial phase of the pandemic, including the first months after the outbreak of the pandemic, changes in symptoms were observed. The symptoms decreased with time and in specific contexts, such as the loosening of restrictions and the onset of the holiday season.

## Figures and Tables

**Table 1:** Descriptive statistics and prevalence of disorders.

	T1		T2		
Variables	M	SD	M	SD	*n*
Adjustment Disorder					
Number of Stressors	5.34	2.25	5.42	2.50	360
Core Symptoms	21.23	6.55	20.11	6.67	330
Accessory Symptoms	31.59	8.88	29.98	9.68	330
Preoccupation	11.33	3.55	10.50	3.56	330
Failure to Adapt	9.90	3.53	9.61	3.63	330
Avoidance	9.94	3.42	9.57	3.67	330
Depressive Mood	8.32	2.56	7.89	2.65	330
Anxiety	5.41	1.86	5.10	1.94	330
Impulsivity	7.92	2.81	7.41	2.87	330
PTSD					
Re-experiencing	2.78	2.36	4.86	2.48	287
Avoidance	3.59	2.53	5.53	2.62	287
Sense of Threat	3.68	2.61	5.37	2.77	287
PTSD Functional Impairment	4.31	3.87	4.07	3.90	287
Depression Symptoms	11.20	6.52	10.05	6.55	279
Symptoms of Generalized Anxiety	9.55	5.87	7.71	5.72	366
Cognitive Attentional Syndrome	75.40	19.88	73.96	20.33	279
Impaired Functioning	21.13	8.59	18.23	10.54	362
Prevalence					
Impaired Functioning					
Mild Functional Impairment	13%	*n* = 215	30%	*n* = 109	
Moderately Severe Functional Impairment	35%	*n* = 601	26%	*n* = 96	
Severe Functional Impairment	52%	*n* = 877	44%	*n* = 161	
Depression	31%	*n* = 520	19%	*n* = 70	
Generalized Anxiety	45%	*n* = 764	33%	*n* = 122	

**Table 2:** Indirect effects.

Effects	T1		T2	
	Estimate		Estimate	
	90% CI	*p*	90% CI	*p*
Stressors –> CAS –> PTSD	0.028 0.006–0.050	0.038	0.070 −0.012–0.152	0.162
Stressors –> AjD –> PTSD	0.112 0.085–0.139	<0.001	0.145 0.071–0.219	0.001
Stressors –> CAS –> AjD –> PTSD	0.153 0.128–0.178	<0.001	0.211 0.137–0.284	<0.001
Stressors –> AjD –> IF	0.104 0.080–0.128	<0.001	0.124 0.061–0.186	0.001
Stressors –> CAS –> AjD –> IF	0.142 0.123–0.161	<001	0.180 0.129–0.231	<0.001
Stressors –> AjD –> Depression Symptoms	0.110 0.083–0.137	<0.001	0.174 0.076–0.272	0.003
Stressors –> CAS –> Depression Symptoms	0.042 0.020–0.065	0.002	0.025 −0.076–0.127	0.681
Stressors –> CAS –> AjD –> Depression Symptoms	0.150 0.124–0.150	<0.001	0.254 0.149–0.359	<0.001
Stressors –> CAS –> GAD	0.041 0.021–0.061	0.001	0.016 −0.059–0.091	0.730
Stressors –> AjD –> GAD	0.120 0.091–0.149	<0.001	0.168 0.079–0.257	0.002
Stressors –> CAS –> AjD –> GAD	0.164 0.139–0.189	<0.001	0.244 0.158–0.330	<0.001

Notes: CAS—Cognitive Attentional Syndrome, AjD—Adjustment Disorder, GAD—Symptoms of Generalized Anxiety, IF—Impaired Functioning.

## Data Availability

The data can be made available by the corresponding authors upon request.
